# A cecal slurry mouse model of polymicrobial abdominal sepsis to evaluate the effects of antiplatelet agents

**DOI:** 10.1016/j.rpth.2025.103292

**Published:** 2025-12-10

**Authors:** Alexandre Mansour, Isabelle Gouin-Thibault, Elisa Rossi, Aurore Marchelli, Tiphaine Belleville-Rolland, Nicolas Nesseler, Pascale Gaussem, Christilla Bachelot-Loza

**Affiliations:** 1 Department of Anesthesia and Critical Care, Pontchaillou, University Hospital of Rennes, Rennes, France; 2 Univ Rennes, CHU Rennes, Inserm, IRSET, UMR_S 1085, Rennes, France; 3 Department of Hematology, Pontchaillou, University Hospital of Rennes, Rennes, France; 4 Université Paris Cité, Optimisation thérapeutique en neuropharmacologie, INSERM U1144, Paris, France; 5 Etablissement Français du Sang, AP-HP, Hôpital Necker-Enfants Malades, Paris, France; 6 Univ Rennes, CHU Rennes, Inserm, CIC 1414, Inra, Inserm, Institut NUMECAN – UMR_A 1341, UMR_S 1241, Rennes, France; 7 Université Paris Cité, INSERM UMR-S970, Paris Cardiovascular Research Center, Paris, France; 8 AP-HP, Service d’hématologie biologique, Hôpital Européen Georges Pompidou, Paris, France

## 1 Introduction

Sepsis is a life-threatening condition caused by a dysregulated host response to infection [1]. Platelets are involved in sepsis, with thrombocytopenia being correlated with prognosis [2]. By releasing inflammatory mediators and interacting with leukocytes, platelets drive immunothrombosis and neutrophil extracellular trap formation [3]. While important for host defense, unregulated platelet activation may promote organ failure.

The effects of antiplatelet agents in sepsis have mostly been studied using the cecal ligation and puncture (CLP) model. This model is considered the gold standard for experimental sepsis but is associated with variability and reproducibility challenges [4,5]. The cecal slurry (CS) model, based on intraperitoneal injection of cryopreserved cecal content, offers a reproducible phenotype of systemic inflammation and organ injury, with low interoperator variability and no surgery [6,7]. However, this model has not yet been used to assess antiplatelet agents, particularly P2Y12 inhibitors.

The ADP P2Y12 receptor is crucial for platelet activation [8]. Clopidogrel, a P2Y12 inhibitor requiring hepatic activation by cytochrome P450, has shown variable efficacy in sepsis [[9], [10], [11], [12], [13]]. This variability likely reflects differences in metabolic activation and platelet responsiveness, both of which may be impaired during sepsis because of altered liver function, inflammation, or drug interactions. In contrast, ticagrelor—a direct-acting P2Y12 inhibitor—reduced thrombo-inflammatory markers in patients with pneumonia [14] and reduced mortality following sepsis [15]. It reduced systemic inflammation in healthy volunteers administered Escherichia coli endotoxin [16] and showed potent efficacy in CLP murine sepsis models [[17], [18], [19]]. Its beneficial effects were also reported in a PLATO trial subanalysis [15].

Our objective was to apply the CS model as a suitable murine model of sepsis to evaluate the effects of ticagrelor, under the hypothesis that it could reproduce the beneficial effects observed in the CLP model. Importantly, this model has never been tested in hemostasis, and we aimed to determine whether the effects of ticagrelor on human sepsis are reproducible in the CS model.

## 2 Methods

### 2.1 CS model of sepsis

Twelve to 16-week-old male C57BL/6JRj mice (Janvier Labs) were housed under controlled conditions with free access to water and food. All procedures were approved by the French Ministry of Research (13799-2017120613101888-V7), in compliance with EU Directive 2010/63/EU for animal experiments. Male mice were selected based on previous evidence that ticagrelor was effective in male but not in female mice in the CLP model [19], consistent with our aim to apply the CS model as an alternative to CLP.

**Figure 1:**
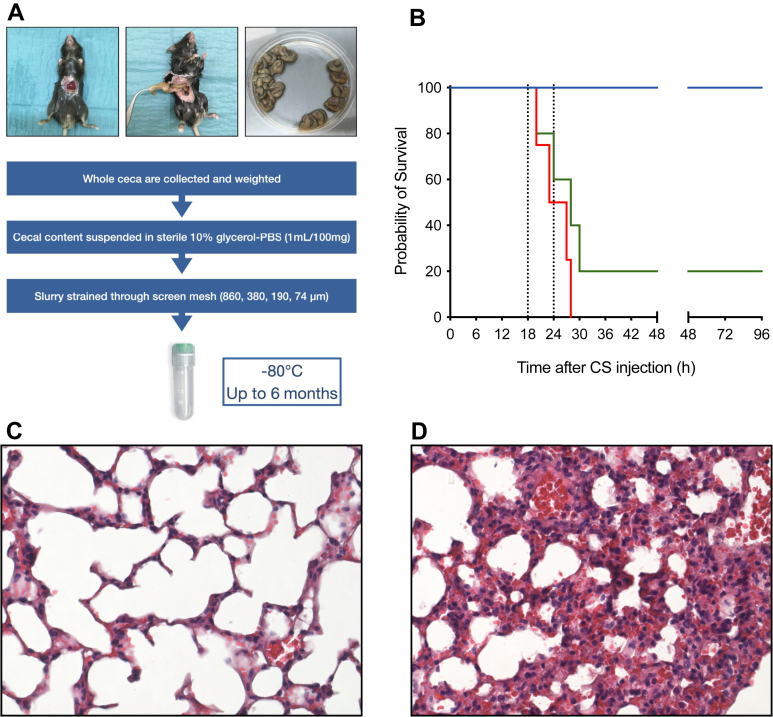
Preparation of cecal slurry (CS) and severity of sepsis after injection. (A) Cecal content from donor mice were suspended in sterile 10% glycerol–phosphate-buffered saline (PBS) with a ratio of 1 mL for 100 mg of wet cecal content weight. The CS was filtered using sterile mesh screens and then dispensed into cryovials (2 mL) under continuous stirring using a magnetic stir bar and stored at −80 °C up to 6 months. (B) Healthy 12- to 16-week-old male C57BL/6JRj mice were injected intraperitoneally either with 400 μL glycerol-PBS (blue solid line, *n* = 4), 300 μL CS (approximately 1 mg/g; green solid line, *n* = 5), or 400 μL CS (approximately 1.3 mg/g; red solid line, *n* = 4). No additional therapeutic intervention was performed. Survival was monitored up to 96 hours. (C, D) Mice were injected intraperitoneally with either 300 μL glycerol-PBS (C) or with 300 μL CS (approximately 1 mg/g; D). Lungs were harvested and fixed 18 hours after CS injection. Lung sections (5 μm) were stained with hematoxylin and eosin and analyzed at ×400 total magnification using a light microscope. (C) Representative stained section of a nonseptic control mouse. (D) Representative stained section of a septic mouse.

CS preparation and injection followed established protocols [7]. Donor mouse cecal content was suspended in 10% glycerol–phosphate-buffered solution (PBS), filtered and stored at −80 °C (Figure 1A). Three independent batches of CS were used and showed an excellent reproducibility in bacterial content (data not shown). Bacterial viability and composition remained stable after 6 months at −80 °C. For sepsis induction, frozen CS aliquots were thawed at 37 °C and injected intraperitoneally using a 25-gauge needle. Control mice received 10% glycerol-PBS (vehicle). No other treatments were given.

Based on the study by Steele et al. [7], who reported 100% mortality at 500 μL and 66% at 400 μL within 48 hours, a preliminary study comparing 300 and 400 μL determined 300 μL as the optimal CS injection volume, ensuring survival up to 96 hours (Figure 1B). The experimental end point was set at 18 hours to capture severe sepsis and organ injury without mortality. Mice (weight, 30 g ± 15%) received 300 μL of either glycerol-PBS (vehicle) or CS (30 mg, approximately 1 mg/g). Survival, murine sepsis score (MSS) [20], and temperature were recorded at H0, H1, and H6 and at the final time point. MSS evaluation was blinded. Mice with MSS over 21 at any time point were euthanized.

### 2.2 Ticagrelor treatment

Ticagrelor (100 mg/kg in 10 mM HCl; Brilique; AstraZeneca) or placebo (10 mM HCl) was administered by oral gavage 1 hour before CS (or vehicle) injection. Effective inhibition of P2Y12 was confirmed in preliminary experiments using VASP assay (CY-QUANT VASP/P2Y12; Biocytex/Stago).

### 2.3 Blood and lung collection

Mice were anesthetized 18 hours after CS (or vehicle) injection by intraperitoneal injection of ketamine (80 mg/kg; Clorketam; Vetoquinol) and xylazine (10 mg/kg; Rompun; Bayer). Then, blood was collected by cardiac puncture into ACD-C solution (13 mM citric acid, 12.6 mM sodium citrate, 11 mM D-glucose; final concentrations). Platelet and white blood cell counts were determined using an automatic cell counter (MS9; MELET SCHLOESING Laboratoires). Plasma samples were obtained by immediately centrifuging blood and stored at −80 °C until use. After euthanasia, lungs were harvested and fixed in 4% paraformaldehyde for 24 hours at 4 °C.

### 2.4 Assessment of inflammatory markers and organ injury

Liver injury was evaluated by plasma levels of aspartate transaminase (AST) and alanine transaminase (ALT; Architect C-16000; Abbott Laboratories). Plasma levels of interleukin (IL)-6, IL-10, and tumor necrosis factor (TNF)-α were assessed using Luminex assay (R&D Systems). Platelet-neutrophil and platelet-monocyte aggregates were quantified by flow cytometry using anti–CD41-FITC and anti–Ly-6G-APC or anti–Ly-6C-APC antibodies (Miltenyi Biotec) on a BD FACSCalibur cytometer (Becton Dickinson). Results were expressed as the percentage of double-positive events among neutrophils or monocytes. Bloodstream infection was assessed by blood culture (BD BACTEC; Becton Dickinson). Fixed lungs were embedded within paraffin, and sections (5 μm) were stained with hematoxylin and eosin and analyzed using a light microscope (400×). Acute lung injury (ALI) was assessed by blinded visual scoring (4 random fields) using the lung injury scoring system [21].

### 2.5 Statistical analyses

Analyses were performed using Prism 9 (GraphPad Software). Not all assays were performed in all animals; panel-specific sample sizes are reported in figure legends. Mixed models or analysis of variance (anova) with correction for multiple comparisons were used for comparisons between groups. Mann–Whitney U-test was used for ALI score comparison between ticagrelor and placebo groups. Values inferior to the limit of quantification were substituted by zero for cytokine measurement. Data are reported as median with IQR or mean with SD.

## 3 Results

### 3.1 CS model induces a severe sepsis phenotype

**Figure 2:**
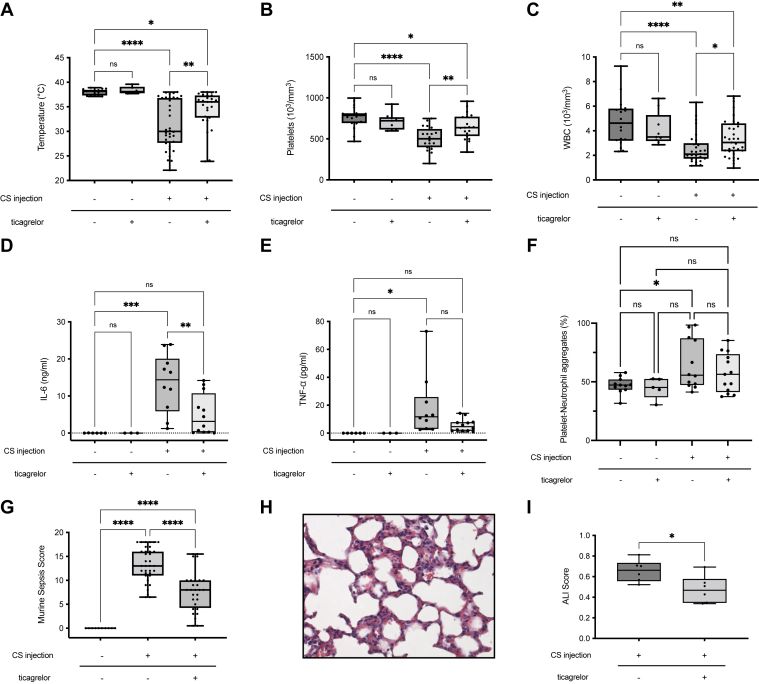
Effect of ticagrelor treatment on cecal slurry (CS)-induced sepsis and acute lung injury (ALI). Mice received ticagrelor (100 mg/kg in 10 mM HCl) or placebo (10 mM HCl) by oral gavage 1 hour before intraperitoneal injection of either 300 μL glycerol–phosphate-buffered saline (PBS; vehicle) or 300 μL CS (approximately 1 mg/g). All parameters were assessed 18 hours after CS (or vehicle) injection. (A) Temperature: vehicle-treated mice (*n* = 6 for placebo; *n* = 18 for ticagrelor) and CS-treated mice (*n* = 36 for placebo; *n* = 27 for ticagrelor) were evaluated for rectal temperature. (B, C) Platelet and white blood cell (WBC) counts: vehicle (*n* = 19 for placebo; *n* = 9 for ticagrelor) and CS (*n* = 19 for placebo; *n* = 20 for ticagrelor) groups were analyzed for blood cell counts. (D, E) Cytokines: plasma inteleukin (IL)-6 and tumor necrosis factor (TNF)-α levels were measured in vehicle (*n* = 6 for placebo; *n* = 6 for ticagrelor) and CS (*n* = 10 for placebo; *n* = 12 for ticagrelor) groups. (F) Platelet-neutrophil aggregates: quantified by flow cytometry using anti–CD41-FITC and anti–Ly-6G-APC antibodies and expressed as the percentage of double-positive events among neutrophils. Vehicle groups included *n* = 11 for placebo and *n* = 5 for ticagrelor; CS groups included *n* = 11 for placebo and *n* = 13 for ticagrelor. (G) Murine sepsis score (MSS): vehicle group (*n* = 10) and CS groups (*n* = 35 for placebo; *n* = 28 for ticagrelor). MSS was evaluated in blinded conditions. (H, I) ALI: ticagrelor (*n* = 6) or placebo (*n* = 6) was administered before CS injection. Lungs were harvested and fixed 18 hours later, sectioned (5 μm), and stained with hematoxylin and eosin (×400 magnification). Lung injury was scored blindly across 4 random fields per mouse using the following criteria: (a) neutrophils in the alveolar space, (b) neutrophils in the interstitial space, (c) hyaline membranes, (d) proteinaceous debris, and (e) alveolar septal thickening. The final injury score was calculated as follows: score = (20 × a + 14 × b + 7 × c + 7 × d + 2 × e)/(number of fields × 100). ∗*P* < .05; ∗∗*P* < .01; ∗∗∗*P* < .001; ns: nonsignificant (*P* ≥ .05).

CS injection induced severe sepsis at 18 hours, with hypothermia (Figure 2A), high MSSs (Figure 2G), and bloodstream infections in all tested mice (n = 6). Lung sections showed neutrophil infiltration, hyaline membranes, proteinaceous debris, and septal thickening (Figure 1D vs C).

CS sepsis was associated with thrombocytopenia (501 [IQR, 398-621] vs 788 [IQR, 693-812] g/L; *P* < .001), leukopenia (2.1 [IQR, 1.7-3.0] vs 4.6 [IQR, 3.2-5.8] g/L; *P* < .001) (Figure 2B, C), and a systemic inflammatory response with increased IL-6 (14.4 [IQR, 5.9-20.1] vs 0 [IQR, 0-0.1] ng/mL; *P* < .001) (Figure 2D), TNF-α (11.7 [IQR, 3.1-25.9] vs 0 [IQR, 0-0] pg/mL; *P* = .037) (Figure 2E), and IL-10 (280.4 [IQR, 84.0-803.1] vs 0 [IQR, 0-0.1] pg/mL; *P* = .016). Soluble P-selectin increased markedly (80.9 [IQR, 61.3-131.5] vs 27.0 [IQR, 24.9-32.2] ng/mL; *P* = .009).

Platelet-neutrophil aggregates were significantly elevated (55.6% [IQR, 47.3%-87.2%] vs 47.3% [IQR, 43.3%-52.0]; *P* = .046) (Figure 2F), as were platelet-monocyte aggregates (56.7% [IQR, 51.6%-63.4%] vs 44.5% [IQR, 38.2%-48.5%]; *P* = .028; data not shown). CS sepsis also induced organ injury, with high ALI scores (Figure 2H, I) and elevated AST (190 [IQR, 172-288] vs 45 [IQR, 44-54] U/L; *P* < .001) and ALT (56 [IQR, 50-71] vs 20 [IQR, 19-20] U/L; *P* < .001).

### 3.2 Ticagrelor treatment reduces functional impact of sepsis and improves thrombocytopenia and leukopenia

Effective P2Y12 inhibition by ticagrelor was confirmed in preliminary experiments (VASP platelet reactivity index, 9% ± 12% vs 42% ± 6%; *P* = .016; n = 4–5). Ticagrelor administration to nonseptic mice had no significant effect (Figure 2).

In CS sepsis, ticagrelor lessened hypothermia (Figure 2A) and MSS (Figure 2G) and improved thrombocytopenia (to 637 [IQR, 534–771] g/L; *P* = .007) and leukopenia (to 3.1 [IQR, 2.3–4.6] g/L; *P* = .028), although values remained less than those of controls (Figure 2B, C).

Ticagrelor significantly reduced IL-6 (3.2 [IQR, 0.2–10.7] ng/mL) (Figure 2D) and improved ALI (Figure 2H, I). TNF-α (4.6 [IQR, 1.7–7.8] pg/mL) (Figure 2E) and IL-10 (230.2 [IQR, 87.8–347.1] pg/mL) tended to decrease but were not significant. Platelet-neutrophil aggregates (Figure 2F) and liver enzymes (AST, 186 [IQR, 120–213] U/L; ALT, 58 [IQR, 49–70] U/L) were unchanged.

## 4 Discussion

In this study, the CS model reproduced key features of severe sepsis, including hypothermia, cytopenia, inflammatory cytokine release, and organ injury. Pretreatment with ticagrelor significantly improved the sepsis phenotype and reduced ALI.

Previous studies suggested that platelet P2Y12 inhibition mitigates inflammation using rodent models of lipopolysaccharide-induced inflammation or CLP-sepsis with clopidogrel or P2Y12 receptor-deficient mice [9,10,22]. However, these studies were limited by sterile inflammation or variable CLP severity. A recent study using the CLP model found no benefit of clopidogrel or platelet-specific P2Y12 receptor deletion [12]. Additionally, clinical observations indicated potential clopidogrel inefficacy, due to its reliance on hepatic metabolism for activation, which may be impaired during sepsis [13,23,24].

In contrast, consistent with our results, ticagrelor showed consistent anti-inflammatory and organ-protective effects in both clinical and experimental studies [[14], [15], [16], [17], [18]]. Ticagrelor also inhibits ENT-1, increasing plasma adenosine, which may further reduce inflammation [3], and additionally, ticagrelor has demonstrated bactericidal activity in a mouse model of implanted Staphylococcus aureus [25]. We therefore used ticagrelor to demonstrate that the CS model can reproduce pharmacologic effects previously observed in other sepsis models.

Our CS preparation protocol ensured reproducible bacterial content, long-term stability, and low interoperator variability [6]. The CS model allowed rapid induction of polymicrobial sepsis without surgical intervention, avoiding the variability inherent to CLP.

Since this study was designed to validate the applicability of the CS sepsis model, it has several limitations concerning the effects of ticagrelor itself. The dose used exceeded standard antithrombotic levels, and only pretreatment was assessed. However, we used similar dose to that proven effective in the CLP model [17]. Biomarkers of immunothrombosis and bleeding risk were not evaluated, nor was the relationship between VASP inhibition and outcomes. These aspects warrant further investigation and may help refine the translational relevance of this model. Future studies should also examine sex-related differences reported in the CLP model [19].

In conclusion, the murine CS model provides a practical and reproducible tool to evaluate pharmacologic interventions such as ticagrelor in polymicrobial sepsis and may facilitate future preclinical testing of anti-inflammatory or antithrombotic therapies.
